# Continuing Professional Development – Radiation Therapy

**DOI:** 10.1002/jmrs.708

**Published:** 2023-08-14

Maximise your CPD by reading the following selected article and answer the five questions. Please remember to self‐claim your CPD and retain your supporting evidence. Answers will be available via the QR code and online at www.asmirt.org/news-and-publications/jmrs, as well as published in JMRS – Volume 70, Issue 4 December 2023.

## Radiation Therapy—Original Article

### Routine prophylactic percutaneous endoscopic gastrostomy in head and neck cancers with bilateral neck irradiation: A regional cancer experience in New Zealand

Fong SC, Pandey R, Rajaretnam M, Delaibatiki M, Peel DNY. (2023). *J Med Radiat Sci*. https://doi.org/10.1002/jmrs.699

1. Which statement best describes the current guidelines regarding the use of prophylactic percutaneous endoscopic gastrostomy **(**PEG) tubes in head and neck cancer patients?
  - Prophylactic PEG tubes are recommended for all head and neck cancer patients.
  - Prophylactic PEG tubes are discouraged due to the high risk of infection.
  - Prophylactic PEG tube placement is made on an individual basis, considering the patient's overall health, treatment plan, and anticipated difficulty with oral intake.
  - Prophylactic PEG tubes are only used for palliative care.
2. What is the Subjective Global Assessment of nutritional status?
  - A tool used to assess nutritional status based on objective measures such as BMI.
  - A tool used to assess nutritional status based on subjective measures such as patient history and physical examination.
  - A tool used to assess tumour characteristics based on imaging studies.
  - A tool used to assess treatment response based on blood tests.
3. What is the role of a multidisciplinary team in cancer care?
  - To provide emotional support for patients during treatment.
  - To evaluate the effectiveness of PEGs.
  - To determine the best treatment regimen for cancer patients.
  - To determine the need for a PEG.
4. According to this study, what was the most frequent complication experienced by patients with PEGs?
  - Peristomal infection
  - Bowel perforation and ileus
  - Significant pain after PEG insertion
  - Mild leakage from the PEG
5. According to the findings of this study, which of the following statements is false?
  - Weight loss and hospitalisation rates among head and neck cancer patients are consistent with previous studies.
  - Prophylactic PEG procedures have high long‐term dependence among head and neck cancer patients.
  - Xerostomia was the main reason for grade 1 and 2 late dysphagia in patients.
  - Chemoradiation therapy was the most common treatment among the patients.

Recommended further reading:1

Petkar
I
, 
Rooney
K
, 
Roe
JW
, 
Patterson
JM
, 
Bernstein
D
, 
Tyler
JM
, 
Emson
MA
, 
Morden
JP
, et al. DARS: a phase III randomised multicentre study of dysphagia‐ optimised intensity‐ modulated radiotherapy (Do‐IMRT) versus standard intensity‐ modulated radiotherapy (S‐IMRT) in head and neck cancer. BMC Cancer. 2016; Oct 6;16(1): 770. doi: 10.1186/s12885-016-2813-0.27716125PMC50529452

Anderson
NJ
, 
Jackson
JE
, 
Smith
JG
, 
Wada
M
, 
Schneider
M
, 
Poulsen
M
, 
Rolfo
M
, 
Fahandej
M
, et al. Pretreatment risk stratification of feeding tube use in patients treated with intensity‐modulated radiotherapy for head and neck cancer. Head Neck. 2018; Oct;40(10): 2181–92. doi: 10.1002/hed.25316
297563893

Bishop
S
, 
Reed
WM
. The provision of enteral nutritional support during definitive chemoradiotherapy in head and neck cancer patients. J Med Radiat Sci. 2015; Dec;62(4): 267–76. doi: 10.1002/jmrs.132.27512573PMC4968562

## Answers

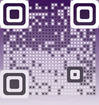

Scan this QR code to find the answers, or visit www.asmirt.org/news-and-publications/jmrs.

## References

[1] Petkar I , Rooney K , Roe JW , Patterson JM , Bernstein D , Tyler JM , Emson MA , Morden JP , et al. DARS: a phase III randomised multicentre study of dysphagia‐ optimised intensity‐ modulated radiotherapy (Do‐IMRT) versus standard intensity‐ modulated radiotherapy (S‐IMRT) in head and neck cancer. BMC Cancer. 2016; Oct 6;16(1): 770. doi: 10.1186/s12885-016-2813-0. 27716125 PMC5052945

[2] Anderson NJ , Jackson JE , Smith JG , Wada M , Schneider M , Poulsen M , Rolfo M , Fahandej M , et al. Pretreatment risk stratification of feeding tube use in patients treated with intensity‐modulated radiotherapy for head and neck cancer. Head Neck. 2018; Oct;40(10): 2181–92. doi: 10.1002/hed.25316 29756389

[3] Bishop S , Reed WM . The provision of enteral nutritional support during definitive chemoradiotherapy in head and neck cancer patients. J Med Radiat Sci. 2015; Dec;62(4): 267–76. doi: 10.1002/jmrs.132. 27512573 PMC4968562

